# Pharmacogenetic analysis of inter-ethnic variability in the uptake transporter *SLCO1B1* gene in Colombian, Mozambican, and Portuguese populations

**DOI:** 10.1186/s12920-023-01642-4

**Published:** 2023-09-01

**Authors:** Mulata Haile Nega, Derbew Fikadu Berhe, Vera Ribeiro

**Affiliations:** 1 Department of Medical Biochemistry, School of Medicine, College of Health Sciences, Mekelle University, Mekelle, Ethiopia; 2 Laboratory of Pharmacogenomics and Molecular Toxicology, Center for Molecular and Structural Biomedicine (CBME/CCMAR), University of Algarve, Faro, Algarve Portugal; 3 University of Global Health Equity, Kigali, Rwanda; 4 Department of Pharmacology and Clinical Pharmacy, School of Pharmacy, College of Health Sciences, Addis Ababa University, Addis Ababa, Ethiopia

**Keywords:** c.521 T > C, Polymorphism, *SLCO1B1*, SNP, Statins

## Abstract

**Background:**

Statin-induced myopathy is reported to be associated with the solute carrier organic anion transporter family member 1B1 gene single nucleotide polymorphism, c.521 T > C. There is no epidemiologic data on this gene polymorphism in several countries. Therefore, this study aimed at assessing the genotype and allele frequencies of the gene variant in three countries.

**Methods:**

This study involved healthy individuals from Colombia, Mozambique, and Portugal. Genomic DNA was isolated from blood samples using the Qiamp DNA Extraction Kit (Qiagen). The isolated DNA was genotyped using novel Polymerase Chain Reaction—Restriction Fragment Length Polymorphism. Microstat and GraphPad QuickCal software were used for the Chi-square test and the evaluation of Hardy–Weinberg equilibrium respectively.

**Results:**

A total of 181 individuals’ blood samples were analyzed. Overall, the TT (74.0%) genotype was the highest and the CC (7.8%) was the lowest. Country wise genotypic frequencies were Colombia 47(70.2%) TT, 12(17.9%) TC and 8(11.9%) CC; Mozambique 47(88.7%) TT, 5(9.4%) TC, and 1(1.9%) CC; and Portugal 40(65.6%) TT, 16(26.2%) TC, and 5(8.2%) CC. The reference (T) allele was highest among Mozambicans (93.4%) compared to Colombians (79.1%) and Portuguese (78.7%). Mozambicans showed statistically significant genotypic and allelic frequency differences compared to Colombians (*p* < 0.01) and Portuguese (*p* < 0.01).

**Conclusions:**

Overall and country-wise, CC genotype was less frequent and it is relatively high for Colombians and Portuguese populations. This finding may imply statins risk–benefit variability associated with CC genotype among these populations that needs further understanding.

## Background

Pharmacogenetics is the study of inter-individual variations in DNA sequence related to drug response [1]. Polymorphism in drug transporters plays a major role in inter-individual differences in drug kinetics. Functional changes in drug transporters can affect the pharmacokinetics, subsequent pharmacodynamics, and toxicological effects of drugs. It can also affect susceptibility to certain diseases [2, 3]. Although detailed information on genetic variability in drug transporter genes is available, our knowledge of identifying those genetic variants that have functional significance and how they contribute to inter-individual variability in drug response is still limited.

Numerous studies have shown that polymorphisms in transporter genes can significantly alter the pharmacokinetics of statins. The risk of statin-induced myopathy is raised by a common genetic variation of the organic anion-transporting polypeptide 1B1 (OATP 1B1), which is encoded by the gene *SLCO1B1*. This protein is a member of the solute carrier organic anion transporter family. Similar to this, genetically compromised ABC G2 (ATP-binding cassette G2) transporter efflux activity causes a significant rise in systemic statin exposure. It’s significant to note that the effects of these genetic polymorphisms vary depending on the statin used. This gives the individualization of lipid-lowering therapy a rational foundation [2–4].

The OATP1B1 is a genetically polymorphic influx transporter expressed on the sinusoidal membrane of human hepatocytes, and it mediates the hepatic uptake of many endogenous compounds and xenobiotics. Studies have demonstrated that OATP1B1 plays a major, clinically important role in the hepatic uptake of many drugs [4].

The *SLCO1B1* gene is located on chromosome 12 (gene locus 12p12). Many single nucleotide polymorphisms (SNPs), both nonsynonymous and synonymous, have been discovered in the *SLCO1B1* gene, and several of these affect transport function in vitro and in vivo. Most of the SNPs associated with altered transport function span the transmembrane domains or extracellular loop 5 of OATP1B1 [4, 5].

Considering that these transporters play a key role in the distribution of many drugs and in the transport of endogenous compounds, such as cholesterol and bile acids, inter-individual variability in disease risk and drug response may be explained by the differential prevalence of genetic variants. In spite of its relevance, some populations have not yet been characterized for the SNP (c.521 T > C) rs4149056 of the *SLCO1B1* gene.

In the present study, we examined the allelic frequencies of the SNP in the *SLCO1B1* gene that may play an important role in drug disposition, in populations from three different ethnic and geographic origins. Namely, native African (Mozambican), Latin American (Colombian), and European (Portuguese) populations which are not frequently the target of pharmacogenetic studies, and this is an important issue when considering the bridging of drug dosages and regimens used in different populations.

## Materials and methods

A DNA template was isolated from blood samples obtained from unrelated, healthy populations of Colombia (*n* = 67), Mozambique from Maputo City (*n* = 53), and Portugal (Caucasian) (*n* = 61) from Southern Portugal, recruited from local medical check-ups. Colombian subjects were from the North-West region, mainly from Antioquia and Chocó departments. One blood sample was obtained from each participant for DNA extraction. Blood sampling and genomic DNA extraction were carried out by taking a 10-mL blood sample from each participant in a tube containing ethylenediaminetetraacetic acid (EDTA) and stored at -20 °C prior to DNA extraction. Genomic DNA was extracted with standard methods (QIAamp DNA Blood Mini Kit; Qiagen, Hilden, Germany). Alternatively, in cases when small amounts of blood were available, DNA was prepared using a quick protocol [5].

### Genotyping

The specific polymorphic variant of the *SLCO1B1* gene, the c.521 T > C SNP analyzed in this study (GenBank accession no. NC_000012.10), was performed by a polymerase chain reaction-restriction fragment length polymorphism (PCR–RFLP) assay. PCR reaction volume was 25µL containing 1 µM of each assay-specific primers, 0.3 mM deoxynucleotide triphosphate (dNTPs) (Promega), 3 mM MgCl_2_ (Promega), 1.5 U Taq polymerase enzyme (Promega), 1 × PCR GoTaq Buffer Mix, water, and ≈ 1 µg genomic DNA. The PCR included 40 cycles at 94 °C for denaturation of the genomic DNA and activation of the Taq Polymerase enzyme, 55 °C for annealing of the primers, and 72 °C for extension.

The PCR assay was performed using Tpersonal Thermocycler (Biometra), and finally, electrophoretic separation on 2% (W/V) agarose gel, with a running time of 90 min at 80 V in 1X TAE buffer (Tris–acetate-EDTA buffer; 40 mM Tris, 20 mM acetate, 1 mM EDTA), and visualization of the gel-separated PCR products with Green Safe (NZYTech) staining under UV light (AlphaImager, AlphaInnotech).

### Primer design

Primer-BLAST tools were used for primer design [6] the sequences deposited into the GenBank (http://www.ncbi.nlm.nih.gov/genbank/) to design pairs of allele-specific forward primers that overlap with the *SLCO1B1* allelic variant and its corresponding single reverse primers. The primers employed in performing the PCR for the analyzed SNP, c.521 T > C, were the forward primer (5’ > 3’); GTTAAATTTGTAATAGAAATGC, and the reverse primer (5’ > 3’); GTAGACAAAGGGAAAGTGATCATA.

### Digestion of the PCR product

Ten μL of PCR products were digested with Bsp1286I (Sdul) restriction enzyme (Thermo Scientific), at 37 °C for 2 h. Digested PCR products were separated by electrophoresis using a 2% agarose gel stained with Green Safe and visualized by a UV transilluminator.

In the 260-base pair (bp) long PCR product of the c.521 T > C SNP obtained from TT homozygotes, the recognition site of the enzyme was missing, resulting in no digestion. In the samples with CC genotype the enzyme recognition site was present, being therefore digested into 153-bp and 107-bp long fragments. In samples with the TC genotype, a mixed pattern was observed, with 153 -bp, 107-bp and 260-bp fragments.

The extracted DNA from a blood sample is amplified using the PCR process followed by electrophoretic separation and visualization of the stained gel-separated DNA fragments under UV light.

### Statistical analysis

Statistical analyses were performed by Microstat (Ecosoft Inc, Indianapolis, IN, USA) and GraphPad Quick Cal (www.graphpad.com) software. Statistical significance was considered at *p* < 0.05. And the evaluation of Hardy–Weinberg equilibrium was done on the analyzed populations using chi-square test [7].

## Results

A total of 181 samples were genotyped for the *SLCO1B1* gene variant, c.521 T > C (V174A) rs4149056, from subjects of different ethnic groups, from Colombian (*n* = 67), Mozambican (*n* = 53) and Portuguese (*n* = 61) populations (Table 1).

**Table 1:** The genotypic and allelic distribution of the *SLCO1B1* exonic polymorphism c.521 T > C (V174A) rs4149056 in healthy Colombian, Mozambican, and Portuguese populations

**Population**	**Subjects**	**Genotypes**			**Alleles**	
		**TT n (%)**	**TC n (%)**	**CC n (%)**	**T n (%)**	**C n (%)**
Colombia	67	47 (70.2)	12(17.9)	8(11.9	106(79.1)	28(20.9)
Mozambique	53	47 (88.7)	5(9.4)	1(1.9)	99(93.4)	7(6.6)
Portugal	61	40 (65.6)	16(26.2)	5(8.2)	96(78.7)	26(21.3)
Total	181	134 (74.0)	33(18.2)	14(7.8)	301(83.2)	61(16.8)

The frequency of homozygote T genotype was observed to be comparable (*p* = 1.000) in Portuguese (65.6%) and Colombian (70.2%), while the Mozambican population showed the highest frequency among the three populations (88.7%), differing significantly from both the Portuguese (*p* < 0.01) and Colombian (*p* < 0.01).The frequency of homozygote C genotype was generally low in all populations. Particularly, the lowest (1.9%) was found in Mozambicans. Whereas the frequency of heterozygous TC genotype was found to be similar/the difference was not statistically significant (*p* > 0.05) in Portuguese (26.2%) and Colombian (17.9%).

The percent allelic frequency of T and C in the c.521 T > C (rs4149056) variant was similar in both Colombian and Portuguese populations. The prevalence of the variant allele that leads to reduced function, corresponds to *circa* 20% of the analyzed alleles in Portugal (20.9%) and Colombia (21.3%), being lower in Mozambique (6.6%).

### Hardy–Weinberg equilibrium

To evaluate if the studied populations obey the Hardy–Weinberg equilibrium, the expected genotypic frequencies were calculated from the allelic frequencies determined experimentally, and then compared to the observed genotypic frequencies. Table 2 shows the results of this analysis for the populations characterized in this study.

**Table 2:** Hardy – Weinberg Equilibrium test of *SLCO1B1* exonic polymorphism; c.521 T > C (V174A) rs4149056 in healthy Colombian, Mozambicans, and Portuguese populations

**Population**	**Genotype**	**Observed** **frequency**		**Expected** **frequency**	***P*-value**
Colombians	TT	47	0.70	0.63	< 0.01^a^
	TC	12	0.18	0.33	
	CC	8	0.12	0.04	
Mozambicans	TT	47	0.89	0.87	0.05 >
	TC	5	0.09	0.12	
	CC	1	0.02	0.00	
Portuguese	TT	40	0.66	0.62	0.05 >
	TC	16	0.26	0.34	
	CC	5	0.08	0.05	

^a^*p* < 0.05; statistically significant difference in observed and expected frequencies

The two-tailed *P*-value for the Colombians is less than 0.05, which indicates that there is a significant difference between the observed and expected values and therefore the analyzed sample of the Colombian population doesn’t follow Hardy–Weinberg equilibrium. Whereas for Mozambique and Portugal populations, the difference between the expected and observed genotypic frequencies is not significant (*p* > 0.05) and the analyzed sample of the two populations follows Hardy–Weinberg equilibrium.

### Comparison of the populations analysed

Alleles distribution for the c.521 T > C SNP in the different populations were compared using a 2 × 2 table of contingency, (Table 3).

**Table 3:** Comparison of the allele distribution observed in Colombian, Mozambican and Portugal populations by their two-tailed *P*-value

	**T n (%)**	**C n (%)**	**Colombian**	**Mozambican**	**Portuguese**
Colombian	106(79.1)	28(20.9)	-	0.002 ^a^	1.000
Mozambican	99(93.4)	7(6.6)	0.002^a^	-	0.002 ^a^
Portuguese	96(78.7)	26(21.3)	1.000	0.002 ^a^	-

^a^*p* < 0.05; statistically significant difference in allele frequencies

From the contingency table, the allele frequencies observed in Colombians are different from the ones observed in Mozambicans, and these differences are statistically significant (*p* < 0.05). However, allele frequencies observed in Colombian are similar to those found in Portuguese, since the observed differences are not statistically significant (*p* > 0.05).

## Discussion

In our study, the most dominant genotype in all populations under study was homozygous c.521TT. Individuals with the homozygote variant genotype c.521CC were high among Colombians followed by Portuguese and Mozambicans.

The overall C allele frequency observed in Colombians (20.9%) is different from the ones observed in Mozambicans (6.6%) and is similar to those found in Portuguese (21.3%). This finding is similar with a population pharmacogenomics study done in the same country, specifically in the Antioquia populations where the C allele of the rs4149056 c.521 T > C SNP is found in higher frequency, showing a negative correlation with African ancestry (5%) and a positive correlation with European ancestry (18%) (29).

The highest frequency of the reference T allele found in Mozambique (93.4%) is in agreement with a previous study in another African ancestry, Tanzanians (97%) [8], Indian-Singapore (94%) [9], and Malays-Singapore (89%) [9]. However, Colombian and Portuguese subjects in this study have relatively lower T allelic frequency compared with reports from Tanzania [8], Indian-Singapore and Malays-Singapore [9]_,_ Europeans: Germany (83%) [8] Roma (83%) [10], Hungarian (81%) [10] Austrian (82.3%) [11], and Finnish populations (80%) [12].

In this study, the Colombian population was not found to follow the Hardy–Weinberg equilibrium. This deviation may occur due to a variety of other causes, such as migration, mutation, natural selection, genetic drift, gene flow, nonrandom mating (inbreeding), or assortative mating [13–15]. It may also mean that evolution has occurred within the population. Because all of these disruptive forces commonly occur in nature, real populations rarely exist under the rigid conditions of the HWE (absence of selection, migration, mutation, etc.). Therefore, it may not affect the above comparisons, rather, genetic discrepancies in nature can be measured as changes from this equilibrium state.

Our finding is in agreement with previous reports that indicated population specificity for *SLCO1B1* gene SNPs [16]. Genetic variants of influx transporters, including the *SLCO1B1* gene, are reported to affect statin efficacy and safety [16–22]. Recent knowledge suggests that the *SLCO1B1* polymorphisms may have particularly important effects on the pharmacokinetic profile of statin drugs (e.g., Atorvastatin) and most studies are mainly focused on the influence of the c.521 T > C polymorphism. One study reported an altered pharmacokinetic profile, including high AUC and C_max_ in atorvastatin in individuals with CC genotype compared to the other genotype group of the SNP [19]. In another study, a significantly larger mean AUC _0-48 h_ was observed in subjects with CC than in subjects with TT and TC genotypes. These findings may imply that reducing OATP1B1 transporter function could reduce atorvastatin hepatic clearance [20]. There are also additional clinical studies that have shown individuals with C allele had increased plasma concentrations of OATP1B1 substrates including pravastatin and repaglinide compared to individuals with T allele [18–21].

Elevated statin concentration associated with the CC and/or TC allele is also reported to cause myopathy, elevated baseline cholesterol synthesis rate, bile acid synthesis, and cardiovascular risks [22, 23]. Basolateral OATP transporters are thought to be mediators of approximately 20% of the hepatic uptake of bile acids [24]. The bile acid concentration in the liver could be decreased as a result of impaired activity of OATP1B1 by limiting the access of bile acids from the portal blood. This functional link between cholesterol homeostasis and OATP1B1 could be through bile acid homeostasis, as the regulation of bile acid synthesis and cholesterol homeostasis are tightly linked.

Individuals carrying the CC (17-fold) and TC (5-fold) genotypes have a higher risk of developing myopathy than individuals with the TT genotype [25]. The relatively high frequencies of low-activity variants (CC and TC) in European ancestry may imply a higher risk of statin-induced myopathy [26].

Therefore, the identification and characterization of these genetic variations may help in the development of *SLCO1B1* genotype-based prescriptions or more personalized drug therapies to achieve the benefits of statin therapy more safely and effectively.

Overall, evaluation of SNP frequencies among different populations with variable ethnic backgrounds is useful as a tool to optimize therapeutics according to variable predicted pharmacokinetics. *SLCO1B1* genotyping may have clinical value for closer monitoring of patients on statin medication. The results obtained in this study may contribute to variations in statin safety profiles within the study population.

To the best of our knowledge, this is the first study in those populations; however, the study population included may not represent ethnic variation within a country and the finding should be interpreted with caution. Investigating haplotypes of the SNP was beyond the scope of our study.

## Conclusions

The CC genotype was less frequent, and it is relatively high for Colombian and Portuguese populations. Our result may indicate disparity in statin safety profiles among the study population. This requires further understanding with more population and geographical coverage.

## Data Availability

The datasets used and/or analyzed during the current study are available from the corresponding author on reasonable request. The sequences used for the primer design are deposited at the GenBank link; http://www.ncbi.nlm.nih.gov/genbank/. And the GenBank accession number of the specific polymorphic variant of the *SLCO1B1* gene, c.521 T > C SNP analyzed in this study, is NC_000012.10.

## Abbreviations

3’UTR: 3’-Untranslated region
ABC: ATP-binding cassette
AUC: Area under the concentration–time curve
AUC_0-t_: AUC from time 0 to t hours
Bp: Base pair
cDNA: Coding deoxyribonucleic acid
CL: Clearance
C_max_: Peak plasma concentration
DNA: Deoxyribonucleic acid
dNTPs: Deoxynucleotide triphosphate
MRT_expo_: Mean residence time
NCBI: National Center for Biotechnology Information
OAT/ OATP: Organic anion transporter
OATP1B1: Organic anion transporting polypeptide 1B1
PCR: Polymerase chain reaction
RFLP: Restriction fragment length polymorphism
SLC/ SLCO: Solute carrier
*SLCO1B1*: Solute carrier organic anion transporter family member 1B1
SNP: Single nucleotide polymorphism
TAE: Tris-Acetate-EDTA Buffer
T_max_: Time to peak plasma concentration

## References

[1] European Medicines Agency. Terminology in pharmacogenetics. EMA; 2002. Available at: https://www.ema.europa.eu/en/documents/scientific-guideline/position-paper-terminology-pharmacogenetics_en.pdf. Accessed 14 Sept 2019.

[2] Ho R, Kim R (2005). Transporters and drug therapy: implications for drug disposition and disease. Clin Pharmacol Ther.

[3] Niemi M (2010). Transporter pharmacogenetics and statin toxicity. Clin Pharmacol Ther.

[4] Tirona R, Leake B, Merino G, Kim R (2001). Polymorphisms in OATP-C: identification of multiple allelic variants associated with altered transport activity among European- and African-Americans. J Biol Chem.

[5] Rudbeck L, Dissing J (1998). Rapid, simple alkaline extraction of human genomic DNA from whole blood, buccal epithelial cells, semen and forensic stains for PCR. Biotechniques.

[6] Ye J, Coulouris G, Zaretskaya I, Cutcutache I, Rozen S, Madden T (2012). Primer-BLAST: a tool to design target-specific primers for polymerase chain reaction. BMC Bioinformatics.

[7] Hardy G (1908). Mendelian proportions in a mixed population. Science.

[8] Saber-Ayad M, Manzoor S, El-Serafi A, Mahmoud I, Abusnana S, Sulaiman N (2018). Statin-induced myopathy *SLCO1B1* 521T>C is associated with prediabetes, high body mass index and normal lipid profile in Emirati population. Diabetes Res Clin Pract.

[9] Melo M, Balanco L, Branco C, Mota-Vieira L (2015). Genetic variation in key genes associated with statin therapy in the Azores Islands (Portugal) healthy population. Ann Hum Biol.

[10] Nagy A, Csilla S, Renata S (2015). Marked differences in frequencies of statin therapy relevant *SLCO1B1* variants and haplotypes between Roma and Hungarian populations. Genetics.

[11] Enko D, Harringer S, Oberkanins C, Pühringer H, Halwachs-Baumann GKG (2018). SLCO1B1 c.521T>C genotyping in the Austrian population using 2 commercial real-time polymerase chain reaction assays: an implementation study. Pharmacology.

[12] Santos P, Gagliardi A, Miname M (2012). *SLCO1B1* haplotypes are not associated with atorvastatin-induced myalgia in Brazilian patients with familial hypercholesterolemia. Eur J Clin Pharmacol.

[13] Abramovs N, Brass A, Tassabehji M (2020). Hardy-Weinberg equilibrium in the large scale genomic sequencing era. Front Genet.

[14] Wang J, Shete S (2012). Testing departure from Hardy-Weinberg proportions. Methods Mol Biol.

[15] Graffelman J, Jain D, Weir B (2017). A genome-wide study of Hardy-Weinberg equilibrium with next generation sequence data. Hum Genet.

[16] Nagar SD, Moreno AM, Norris ET, Rishishwar L, Conley AB (2019). Population pharmacogenomics for precision public health in Colombia. Front Genet.

[17] Daka A, Dimovski A, Kapedanovska A (2015). Effects of single nucleotide polymorphisms and haplotypes of the *SLCO1B1* gene on the pharmacokinetic profile of atorvastatin in healthy Macedonian volunteers. Pharmazie.

[18] Pasanen MK, Fredrikson H, Neuvonen PJ, Niemi M (2007). Different effects of *SLCO1B1* polymorphism on the pharmacokinetics of atorvastatin and rosuvastatin. Clin Pharmacol Ther.

[19] Aquilante C, Bushman L, Knutsen S, Burt L, Rome L, Kosmiski L (2008). Influence of *SLCO1B1* and CYP2C8 gene polymorphisms on rosiglitazone pharmacokinetics in healthy volunteers. Hum Genomics.

[20] Kivistö KT, Niemi M (2007). Influence of drug transporter polymorphisms on pravastatin pharmacokinetics in humans. Pharm Res.

[21] Niemi M, Backman J, Kajosaari L (2005). Polymorphic organic anion transporting polypeptide 1B1 is a major determinant of repaglinide pharmacokinetics. Clin Pharmacol Ther.

[22] Kalliokoski A, Neuvonen M, Neuvonen P, Niemi M (2008). Different effects of *SLCO1B1* polymorphism on the pharmacokinetics and pharmacodynamics of repaglinide and nateglinide. J Clin Pharmacol.

[23] Voora D, Shah S, Spasojevic I (2009). The *SLCO1B1*5* genetic variant is associated with statin-induced side effects. J Am Coll Cardiol.

[24] Pasanen M, Neuvonen P, Niemi M (2008). Global analysis of genetic variation in *SLCO1B1*. Pharmacogenomics.

[25] SNPedia: a wiki supporting personal genome annotation, interpretation and analysis. rs4149056. SNPedia. 2019. Available at: https://www.snpedia.com/index.php/Rs4149056. 10.1093/nar/gkr798 PMC3245045 22140107

[26] Ho R, Choi L, Lee W (2007). Effect of drug transporter genotypes on pravastatin disposition in European- and African-American participants. Pharmacogenet Genomics.

[27] Cavaco I, Pedro Gil J, Gil-Berglund E (2003). CYP3A4 and MDR1 alleles in a Portuguese population. Clin Chem Lab Med.

[28] Cavaco I, Reis R, Pedro Gil J (2003). CYP3A4*1B and NAT2*14 alleles in a native African population. Clin Chem Lab Med.

[29] Fernandes N, Figueiredo P, do Rosário VE (2007). Analysis of sulphadoxine/pyrimethamine resistance-conferring mutations of Plasmodium falciparum from Mozambique reveals the absence of the dihydrofolate reductase 164L mutant. Malar J.

